# Surface Lattice Resonances in 3D Chiral Metacrystals for Plasmonic Sensing

**DOI:** 10.1002/advs.202206930

**Published:** 2022-12-27

**Authors:** Mariachiara Manoccio, Vittorianna Tasco, Francesco Todisco, Adriana Passaseo, Massimo Cuscuna, Iolena Tarantini, Giuseppe Gigli, Marco Esposito

**Affiliations:** ^1^ CNR NANOTEC Institute of Nanotechnology Via Monteroni Lecce 73100 Italy; ^2^ Department of Mathematics and Physics Ennio De Giorgi University of Salento Via Arnesano Lecce 73100 Italy

**Keywords:** chirality, circular dichroism, chiral photonics, chiral surface lattice resonances, focused ion beam induced deposition, plasmonic sensing

## Abstract

Chiral lattice modes are hybrid states arising from the chiral plasmonic particles assembled in ordered arrays with opportune periodicity. These resonances exhibit dependence on excitation handedness, and their observation in plasmonic lattices is strictly related to the chiroptical features of the fundamental plasmonic unit. Here, the emergence of chiral surface lattice resonances (c‐SLRs) is shown in properly engineered arrays of nanohelices (NHs), fully three dimensional (3D) chiral nano‐objects fabricated by focused ion beam processing. By tuning the relative weight of plasmonic and photonic components in the hybrid mode, the physical mechanism of strong diffractive coupling leading to the emergence of the lattice modes is analyzed, opening the way to the engineering of chiral plasmonic systems for sensing applications. In particular, a coupling regime is identified where the combination of a large intrinsic circular dichroism (CD) of the plasmonic resonance with a well‐defined balance between the photonic quality factor (Q factor) and the plasmonic field enhancement (M) maximizes the capability of the system to discriminate refractive index (RI) changes in the surrounding medium. The results lay the foundation for exploiting CD in plasmonic lattices to high performance refractometric sensing.

## Graphical abstract

C‐SLRs are hybridized modes selectively excited by circularly polarization handedness, arising from the coupling between the diffractive orders (DOs) of the periodic lattice and the nanostructures plasmonic resonances. By tuning the lattice periodicity, and consequently the plasmonic/photonic fraction, it is possible to maximize the sensitivity to the RI surrounding environment changes, foreseeing novel highly sensitive detection schemes.

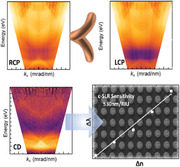

## 1 Introduction

In the last years, Surface Lattice Resonances (SLRs), hybrid states arising from collective excitation of plasmonic nanostructures in periodic lattices, emerged as a promising research field in nanophotonics.^[^ ^1^, ^2^ ^]^ Because of their strong and spectrally narrow optical response, SLRs have been studied as platforms for exploring light‐matter interaction phenomena^[^ ^3^ ^]^ or applied to color filters,^[^ ^4^, ^5^ ^]^ light‐emitting devices,^[^ ^6^, ^7^ ^]^ lenses,^[^ ^8^ ^]^ and nonlinear devices.^[^ ^9^, ^10^, ^11^ ^]^

A new paradigm for these platforms lays in the inclusion of chiroptical effects in the lattice,^[^ ^12^, ^13^ ^]^ leading to the recent observation of c‐SLRs^[^ ^14^ ^]^ and extrinsic chiral effects,^[^ ^15^ ^]^ with the potential to extend the optical properties gamut of hybrid lattice resonances. C‐SLRs arise when the interaction between the resonant chiral localized plasmon (c‐LP) in the unit cell and the in‐plane diffractive orders or Raleigh anomalies (RAs)^[^ ^15^, ^16^, ^17^ ^]^ overcomes the plasmon losses, entering the strong coupling regime. In this condition, the energy detuning between the individual modes defines the plasmonic/photonic contribution to the hybrid state. Such a concept holds the potential for advancing existing schemes of optical detection based on CD,^[^ ^18^, ^19^ ^]^ since other parameters can come into play, namely, the high Q factor of the DOs, the large near‐field amplification M, and the low mode volume (V) of plasmonic nanoantennas.^[^ ^20^, ^21^, ^22^, ^23^ ^]^

NH), as fully 3D nanostructures with chiral shape, exhibit a very large optical activity, even under normal incidence conditions.^[^ ^24^ ^]^ Indeed, in our recent works we described how ordered arrays of 3D NHs can build up a chiral metacrystal, with resulting chiroptical response ruled out by out‐of‐plane and in‐plane lattice parameters,^[^ ^25^ ^]^ and how they can work as biomolecule sensing systems.^[^ ^19^ ^]^ Nevertheless, the generation of c‐SLRs in such a metamaterial has not been fully investigated.

In the present work, we show the emergence of c‐SLRs in a nanohelix‐based metacrystal, and we study the optical properties of the resulting resonances as a function of the energy detuning between plasmonic and diffractive components. As a proof of concept, we discuss the applicability of such platform for sensing applications: combining experimental and theoretical analysis, we show how the system sensitivity is governed by the plasmonic/photonic fraction of the c‐SLR, as a trade‐off between the large Q factor of the diffractive component and the large M of the plasmonic counterpart, reaching an overall sensitivity of 530 nm/RIU. Such an interplay between chirality and SLRs can represent a novel concept for integrated optical nanosensors.

## 2 Results and Discussion

**Figure 1:**
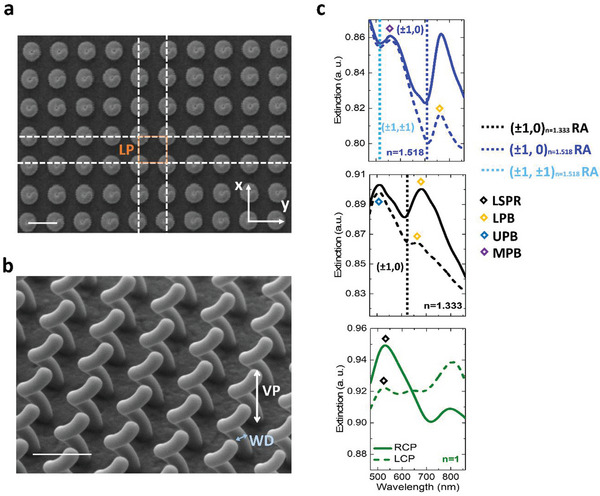
a) Top view SEM image of the NHs array with in‐plane lattice period of 460 nm and 30 × 30 elements. The scale bar is 500 nm. b) SEM image with side view of the NHs array. The vertical pitch (VP) is VP = 500 nm and the wire diameter (WD) is 100 nm. The scale bar is 500 nm. c) Left‐ (dashed line) and right‐handed (continuous lines) circularly polarized extinction spectra of the array in air (*n* = 1, green lines), water (*n* = 1.3334, black lines) and oil (*n* = 1.518, blue lines). The black symbols identify the helix localized plasmon. The DOs are indicated with dashed lines: black (±1,0) for *n* = 1.3334, blue and light blue (±1) ,±1) for *n* = 1.3518, respectively. The upper (UPB), middle (MPB), and lower (LPB) polariton branches of the c‐SLRs are indicated by the navy, violet, and yellow diamonds, respectively.

The fundamental unit of our system is a single, right‐handed (RH), platinum (Pt)‐based nanohelix with an external diameter of 300 nm and vertical pitch of 500 nm, realized by focused ion beam processing^[^ ^26^, ^27^ ^]^on an indium tin oxide (ITO)‐coated glass substrate and replicated several times to form an ordered square array (**Figure** **1**a,b, details in caption). The chiroptical response of the single NH is related to a more efficient excitation of electric dipoles when the circularly polarized light (CPL) matches the structure handedness. The chiral dipoles give rise to collective interactions between neighboring helices, and to a selective extinction of CPL in the visible spectral range, with two opposite CD bands.^[^ ^25^, ^27^ ^]^ Figure 1c shows the extinction spectra of a 30 × 30 elements array with lattice period (LP) of 460 nm, under illumination with CPL at normal incidence, as collected in different refractive index (*n*) media, in particular, air (*n* = 1), DI water (*n* = 1.3334) and oil (*n* = 1.518). When measured in air (bottom panel), the extinction spectra show the fingerprints of a chiral LSPR, with a higher extinction for right‐handed circularly polarized (RCP) light (matching the handedness of the helices), peaked at *λ*_RCP_ = 528 nm. On the other hand, extinction is lower and less structured when interacting with the opposite handedness CPL, with a slightly blue‐shifted peak. In both cases, the large spectral broadening can be inferred to the mixed material composition of the NH.^[^ ^27^ ^]^

When switching to water and oil, the optical impedance mismatch between the superstrate and substrate around the nanostructures is reduced, thus sustaining the radiative coupling among neighbor NHs and increasing the visibility of the DOs,^[^ ^1^, ^28^ ^]^ as shown in Figure 1c (central and upper panel, respectively). At the same time, the increased dielectric constant of the environment induces a redshift of the plasmonic resonance^[^ ^29^ ^]^ toward the energy of the orders of diffraction excited at normal incidence, whose momentum‐energy dispersion for a 2D squared lattice in an homogeneous medium is given by:

(1) $$\lambda_{\mathrm{RA}}=\frac{2\pi n}{\sqrt{{\left(k_{x}+N_{x}\frac{2\pi }{LP}\right)}^{2}+{\left(k_{y}+N_{y}\frac{2\pi }{LP}\right)}^{2}}}$$

where *k*_x_ and *k*_y_ are the k‐vector components of the incident light (*k*_x_ = *k*_y_ = 0, for normal incidence), *N*_x_ and *N*_y_ represent the diffraction orders in the xy basis and *n* is the environment refractive index.^[^ ^30^ ^]^ As further discussed later and shown in Figure 1c, moving from air to water environment, the main RCP resonance redshifts, crosses the first diffractive order at *λ*(1,0) = 610 nm and splits in two peaks, which can be attributed to the upper and lower polariton (UPB and LPB, respectively) branches of an SLR, with UPB = 500 nm and LPB = 660 nm. An analogue trend is observed for the opposite polarization handedness, but with less intense features, as a result of the chiral optical behavior of the single NH, establishing a circular polarization dependent excitation of the SLR, which we can define as a chiral SLR (c‐SLR).

Within oil (Figure 3b, blue line, upper panel), a further redshift of the main spectral features of the c‐SLR is observed, together with the emergence of the higher (±1,±1) RA at 510 nm. The spectral features are sharper and more visible, as compared to the water environment, because of the further reduced RI mismatch between superstrate and substrate.

Since it is known how the array size limits the possibility to observe SLRs,^[^ ^31^, ^32^ ^]^ in Figure S1 (Supporting Information), we also show how the c‐SLR intensity is significantly reduced when switching to a smaller array of 20 × 20 elements, corresponding to a patterned area of 100 µm^2^. The effect of increased size, up to 42 × 42 elements is also discussed in Section S1 (Supporting Information) and reported in Figure S1 (Supporting Information).

**Figure 2:**
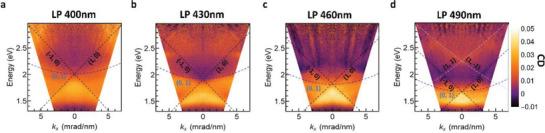
Energy‐momentum experimental CD extinction maps defined as the difference between measured LCP and RCP extinction (shown in Figure S3, Supporting Information) at different LPs (400 nm, 430 nm, 460 nm, 490 nm)., a, b, c, d, respectively). The dashed lines are drawn to highlight the progressive DO dispersion shift when increasing LP. The DOs dispersions were calculated from Equation 1 for the indicated (Nx,Ny) indices.

To shed light on the optical behavior of NHs lattices, we realized arrays with different LPs (400, 430, 460, 490 nm). We experimentally measured the energy‐momentum extinction maps by imaging the transmitted Fourier space under interaction with Left‐handed circularly polarized (LCP) and RCP light, in oil environment. The corresponding CD spectra, calculated as the difference between LCP and RCP extinction, are shown in **Figure** **2** (all the extinction spectra and Fourier space maps are reported for completeness in the Figures S2 and S3, Supporting Information). Interestingly, although the orders of diffraction are linearly polarized, all the CD maps show the emergence of linear and parabolic dispersions directly related to the diffraction modes excited in the lattice. In particular, for LP = 400 nm, a clear band bending can be observed at the (±1,0) RA due to the chiral LSPR of the NHs (Figure 2; Figure S3, Supporting Information). By increasing LP, the hybrid mode continues to be visible and becomes narrower. For the largest LP (490 nm), also higher DOs appear, i.e., the (±1,±1). As discussed above, RCP‐SLRs appear more pronounced than LCP‐SLRs (Figure S3, Supporting Information), because of a more efficient excitation of the NH plasmon with RCP light.^[^ ^27^ ^]^ This behavior is confirmed in Figure S4 (Supporting Information) by the simulated far‐field extinction maps for LCP and RCP, in good agreement with the optical measurements.

The experimentally measured spectral features (Figure 1) and the angular dispersion (Figure 2; Figure S3, Supporting Information) demonstrate the excitation of c‐SLR in the visible range, arising from a properly engineered array of fully 3D chiral NHs. In particular, the proposed system exhibits a large value of maximum CD (*E*_LCP_‐*E*_RCP_ larger than 5%) at the c‐SLR energy (≈800 nm).

To shed light on the optical behavior of our system, we numerically calculated the normal incidence (*k*_x_ = *k*_y_ = 0) extinction of an infinite NHs array as a function of the lattice period and of the incident circular polarization in water environment, of significance for biosensing application. Such a study will show later how the detuning among plasmonic and DOs, controlled by the lattice period, affects the overall system sensitivity.

**Figure 3:**
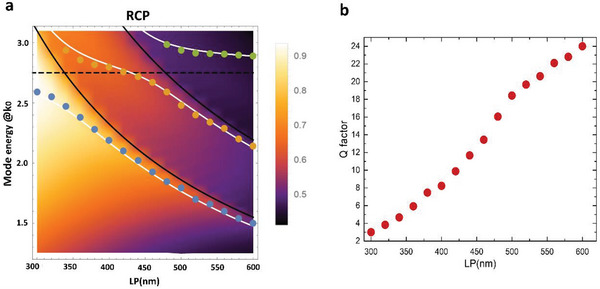
a) Extinction dispersion at normal incidence for RCP light in water environment obtained from numerical simulations as a function of LP (from 300 to 600 nm). The solid black line indicates the calculated LSPR, the dashed black line indicates the calculated dispersions while the white solid lines plot the fitting of the calculated c‐SLR peaks through the three coupled‐oscillator model given by Equation 2, associated to the lower (blue points), middle (orange points), upper (green points) branches. b) Calculated Q factor $\left(\frac{\lambda }{\Delta \lambda }\right)$ for the simulated first order c‐SLR, as a function of the lattice period for RCP polarization.

In order to estimate the bare chiral LSPR (c‐LSPR) position and to fully explore the diffractive coupling mechanism, we started the simulations from a very low LP value (300 nm) corresponding to NHs in closely packed configuration. Here, the reduced LP induces near‐field coupling among adjacent NHs enhancing scattering and leading to stronger effective damping.^[^ ^33^, ^34^ ^]^ Therefore, the consequently larger plasmon mode results not suitable for sensing. Indeed, in the map of RCP case, the c‐LSPR (**Figure** **3**a) appears as a broad peak at around 2.7 eV (dashed horizontal black line). The energy dispersions of the DOs are indicated with solid black lines, as calculated from Equation 1.

By increasing the LP, as the first DO the energy crosses the c‐LSPR (LP = 350 nm), an anti‐crossing clearly appears in the LP‐dependent map for both RCP (Figure 3a) and LCP (Figure S5d, Supporting Information) light. The same behavior is observed at LP = 490 nm, when the LSPR crosses the second order (±1,±1) RA.

For each LP, we extracted the energy position of the extinction peaks by Gaussian profile fitting (represented as coloured dots in the maps). The obtained dispersion and anti‐crossing behavior, arising from the hybridization between the c‐LSPR and the DOs, can be described by a three coupled oscillators model according to the following Hamiltonian:

(2) $$\begin{array}{c} \hat{H}=\left(\begin{array}{ccc} E_{DO\left(\pm 1,0\right)}-i\gamma_{\left(\pm 1,0\right)} & 0 & g_{\left(\pm 1,0\right)}^{P}\\ 0 & E_{DO\left(\pm 1,\pm 1\right)}-i\gamma_{\left(\pm 1,\pm 1\right)} & g_{{\left(\pm 1,\pm 1\right)}_{2}}^{P}\\ g_{\left(\pm 1,0\right)}^{P} & g_{\left(\pm 1,\pm 1\right)}^{P} & E_{c-LSPR}^{P}-i\gamma_{c-LSPR} \end{array}\right) \end{array}$$

Here, *P* represents the light polarization (LCP or RCP), the diagonal elements represent the diffractive wave energies of the (±1,0) and (±1, ±1) DOs, and the c‐LSPR energy, while the off‐diagonal elements, g^P^_(±1,0)_ and g^P^_(±1,±1)_, describe the coupling strength between the corresponding modes. *γ*_*LSPR*_, *γ*_( ± 1, 0)_, *γ*_( ± 1, ±1)_ represent the plasmonic, ( ± 1, 0) and ( ± 1, ± 1) DOs losses respectively, while coupling between different DOs can be ignored .^[^ ^30^ ^]^ In particular, the DO losses hover around a few ten of meV and they are negligible with respect to the c‐LSPR ones.^[^ ^17^ ^]^ The plasmon energy *E*_*c* − *LSPR*_ and the parameters *g^P^* and *γ* have been considered as fitting parameters.

By fitting the peaks position with the Hamiltonian solution, we found for RCP excitation, *E* ^RCP^LSPR = 2,7 eV, *g* ^RCP^_(±1,0)_ ≈340 meV and *g* ^RCP^_(±1,±1)_ ≈300 meV . The obtained branches dispersions are shown in Figure 3a as white dashed curves. Corresponding LCP data are reported in Figure S5d (Supporting Information).

The c‐SLR is a hybrid mode with variable plasmonic/photonic content depending on the coupling conditions tuned by the LP. This can be visualized through the Hopfield coefficients calculated for the various branches observed in the simulated extinction maps of RCP and LCP light (Figure S5, Supporting Information).

To better evidence the photonic behavior of the hybrid mode, in Figure 3b we show that for the Q factor increases as detuning increases, a trend that is also followed by the experimental values calculated in the nanofabricated arrays and reported in Figure S6 (Supporting Information).

**Figure 4:**
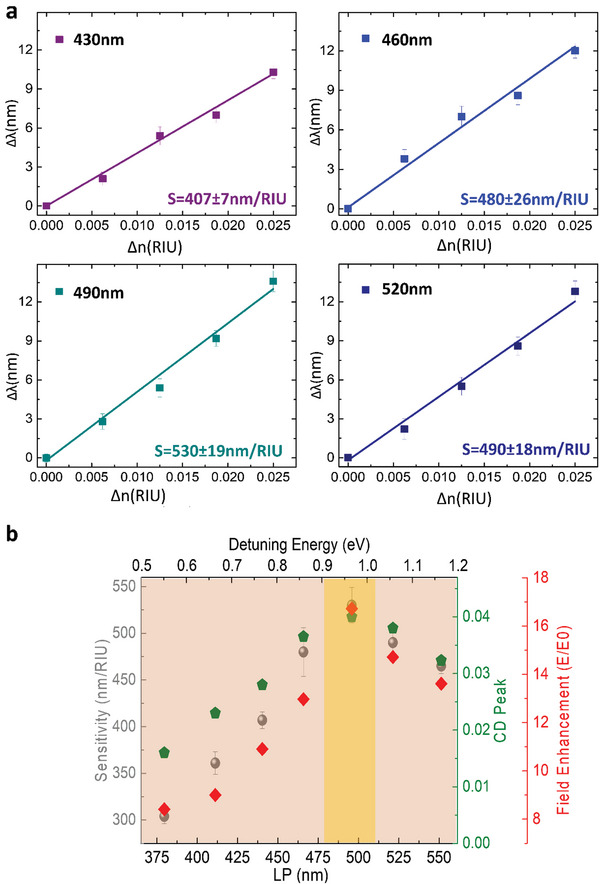
a) Trend of the spectral peak shift versus RI variations for nano‐helices arrays with different LPs immersed in a glycerol‐water solution at different molar concentrations. The line corresponds to the linear fits of the measured spectral positions. The sensitivity, measured as *S* = ∆*λ*/∆*n*, is retrieved by the linear fit. b) Correlation between the sensitivity S the maximum peak intensity of the CD (corresponding to the c‐SLR peak) and the field enhancement M. All the parameters increase up to a maximum value found at LP 490 nm (corresponding to a 0,95 eV detuning between *E*_LSPR_ and *E*_g_^LP^) and then decrease.

In our previous work,^[^ ^19^ ^]^ we exploited 3D core‐shell chiral metamaterials for biomolecular sensing detection at femtomolar concentrations. The intrinsic chirality induced circular polarization dependent optical response with CD spectra free from background interference, allowing to work even in the complex matrix of human serum. Here, as a benchmark also of the proposed c‐SLR based system towards sensing applications, we recorded the energy position of maximum CD in known RI environment (glycerol‐water mixtures from 0% to 20% corresponding to a RI range between n=1.333 and n=1.358^[^ ^35^ ^]^) for the fabricated samples with different lattices. This spectral feature linearly shifts with the RI (**Figure** **4**a; Figure S7, Supporting Information) providing a sensitivity value, *S*, defined as Δ*λ*/Δ*n* (where Δ*λ* represents the wavelength peak shift and Δ*n* the change of the RI of the glycerol‐water solution).

As shown in Figure 4a, the sensitivity S evolves from 360 nm/RIU for small LP, up to the maximum value of 530 nm/RIU for LP = 490 nm and then decreases again to 490 nm/RIU for higher LP. Figure 4b highlights the direct correlation between S and CD experimental trends as a function of LP. The S trend could be explained considering the evolution of the plasmonic contribution to the hybrid mode. We calculated the electric field enhancement, as a function of the detuning energy, averaged at three different points along the nanohelix z‐evolution, in particular at the bottom, center and top side (see Figure S8, Supporting Information). In the short LP region, the strong coupling between the plasmonic and photonic modes leads to the resonance splitting and to the suppression of radiative efficiency at such energy.^[^ ^36^ ^]^ In this condition, characterized by a large plasmonic fraction, there is a large EM field confinement, leading to small modal volume , but not enough to balance the low Q factor (Figure 3b), thus resulting in low M and less efficient sensing (Figure 4b).

On the other hand, for larger detuning (large LP) the Q factor increases due to a more photonic behavior (Figure 3b).^[^ ^36^, ^37^ ^]^

Therefore, to maximize S, it is necessary to find an optimum balance between small chiral plasmonic *V* and large DOs Q factor (both influencing theM value proportional to $Q/\sqrt{V}$ ^[^ ^22^ ^]^) in the c‐SLR (Figure 4b; Figure S5, Supporting Information). A sufficiently low plasmonic fraction can ensure the non‐null and asymmetric scattering with respect to CPL, favoring a large S is achieved in combination with large CD value. Further increment of the LP>490 nm inverts the trend, toward a reduction of the S, because despite of the increment of the Q factor (Figure 3b), the further reduction of the plasmonic fraction corresponds to a low M.^[^ ^22^, ^30^ ^]^ Following these results, we defined an intermediate LP region where the optimized *Q*/*V* ratio allows to reach high sensitivity S in the system.

In order to position our c‐SLR sensor with current state of art in photonic and plasmonic metasensors,^[^ ^38^ ^]^ even though a wide plethora of architectures can be considered, we can make a comparison with some high sensitivity systems (to the best of our knowledge) based either on periodic array of nanostructures exhibiting surface lattice resonance (*S* = 357 nm/RIU,^[^ ^39^ ^]^ *S* = 385 nm/RIU^[^ ^40^ ^]^) or on chiral nanostructures in solution (i.e., not periodically ordered) with CD (*S* = 1091 nm/RIU, in the near‐infrared).^[^ ^18^ ^]^ The obtained results support a large potential held by c‐SLRs for high S purposes, especially considering the possible improvement to the technology related to material composition,^[^ ^41^, ^42^, ^43^ ^]^ helix architecture,^[^ ^44^ ^]^ or even the more challenging array size.

## 3 Conclusion

To conclude, we have shown the coupling between diffractive modes and chiral plasmonic resonances within a periodic array of fully 3D NHs. We have demonstrated that c‐SLR can be excited in the visible spectral range on this system, provided that the criteria of sufficient size, homogeneous RI environment and lattice design are fulfilled. The coupling regime has been studied both theoretically and experimentally, as a function of the LP that regulates the features and the dispersions of CPL extinctions, as well as the relative percentage of photonic and plasmonic mode in the hybridized c‐SLR. The sensing capability of the system has been also studied, with respect to surrounding medium RI tracking through the CD spectra. It is found that the sensitivity to RI changes in the environment can be maximized thanks to a trade‐off between the photonic nature and the plasmonic losses in the hybrid mode (c‐SLR). In particular, a small plasmonic fraction of the hybrid mode is desirable to preserve the chiro optical features of the system. Along with biosensing applications, the investigated mechanism can have several implications in miniaturized and integrated photonics, especially with respect to polarization control in optical emission.

## 4 Experimental Section

### Sample Fabrication

The pt‐based NHs arrays had been grown on an ITO‐on‐glass substrate by means a Carl Zeiss Auriga40 Crossbeam FIB/SEM system coupled with a gas injection system (GIS) and trimethyl(methylcyclopentadienyl)platinum(iv) had been used as gaseous precursor. The helix nanofabrication was obtained by Ga+ beam with energy at 30 keV, the beam current at 1 pA, and the step size, at 10 nm. The chamber pressure was kept between 8 × 10^−7^ mbar and 1.06 × 10^−6^ mbar during the process. 900 helices are replicated as in^[^ ^25^ ^]^ according a predefined lattice design. During the growth, the proximity effects and the local pressure variation, that can affect the final size of each structures, had been controlled inserting a refresh time of 5 min each 30 elements, in order to keep the same values in the vacuum chamber and avoid the pressure drop.

### Optical Characterization

Extinction spectra both in real and the Fourier space imaging were recorded by using a homemade confocal setup made of an optical microscope Zeiss Axioscope A1 with a spectrometer. The sample was illuminated by CPL and focalized with a condenser with variable numerical aperture (from NA<0.1 to 0.95). The light was then collected using a 40x, NA = 0.95 objective lens. Subsequently, the light passes through a three lenses system: the first reconstructs the real space, the second collimates the light beam, and the third lens refocuses the image in the real space. The light was then directed to a Hamamatsu Orca R2 CCD camera and a 200 mm spectrometer. By using all the three lenses combined with adjustable squared slits, the image can be selected in space. Removing the intermediate lenses, instead, Fourier space imaging had been obtained. The circularly polarized light had been produced using a linear Polarizer (Carl Zeiss, 400–800 nm) and a superachromatic waveplate (Carl Zeiss, 400–800 nm).

### Mode Fitting

The maximum values of the normal incidence extinction and CD spectra were extracted by multiple Lorentzian fittings. The dispersions were fitted by using the solution for the eigenvalue problem of the Hamiltonian in Equation 2 for both incident circular polarizations and CD.

### Numerical Simulations

Numerical simulations were performed by exploiting FTDT Based software. A single nanohelix was considered and employed periodic boundary (Block) conditions in the array directions both to simulate extinction spectra and for energy−momentum extinction maps E(*k*_x_,*k*_y_ = 0) for different lattice periods. For the optical properties of the nanohelix, a effective permittivity dispersion of platinum/carbon (Pt/C) alloy calculated in the previous work was considered.^[^ ^27^ ^]^ The ITO/glass substrate was not included in the simulations. The nanohelices had been considered in a nondispersive dielectric media with different refractive indices.

## Conflict of Interest

The authors declare no conflict of interest.

## Supporting information

Supporting Information

## Data Availability

Research data are not shared.
