# Supplementary material for: Surface Lattice Resonances in 3D Chiral Metacrystals for Plasmonic Sensing
Source: Adv Sci (Weinh). 2022 Dec 27;10(6):2206930. doi: 10.1002/advs.202206930 (PMC9951338; doi:10.1002/advs.202206930)
Supplement: Supplementary file 1 — Supporting Information [file ADVS-10-2206930-s001.pdf]

# Supporting Information

## Surface Lattice Resonances in 3D Chiral Metacrystals for Plasmonic Sensing

*Mariachiara Manoccio[1]‖, Vittorianna Tasco[1]*‖, Francesco Todisco[1], Adriana Passaseo[1], Massimo Cuscuna[1], Iolena Tarantini[2], Marco Esposito[1]**

1. CNR NANOTEC Institute of Nanotechnology, Via Monteroni, 73100 Lecce, Italy
2. Department of Mathematics and Physics Ennio De Giorgi, University of Salento, Via Arnesano, 73100 Lecce, Italy

*** Corresponding authors e-mail: vittorianna.tasco@nanotec.cnr.it; marco.esposito@nanotec.cnr.it.**

**‖ these *authors contributed equally* to the *work***

### S1. Effect of the Array Size

In order to give rise to the c-SLRs, another criterion is represented by the element number of the array[1,2]. In figure S1, we show how the observation of chiral SLR is reduced with small number of elements. We report LCP and RCP spectra with 20x20 elements (for a patterned area of 100 μm$^2$ ), 30x30 elements (for a patterned area of 225 μm$^2$) and 42x42 elements (for a patterned area of about 400μm$^2$). The nanofabrication of precise 3D structures is still a challenging nanofabrication issue. Our approach involves FIBID technology suitable to develop, in a bottom-up single direct writing step, more complex and fully 3D structures with high precision and reproducibility. However, FIBID accuracy is affected by the large area scalability because of local pressure variations and proximity effects. Consequently, increasing the number of elements, also the optical response can be affected since small lateral displacements and side deviations owed to fabrication tolerances can broaden the spectral features[3,4]. For this reason in our further considerations we considered arrays with 30x30 elements.

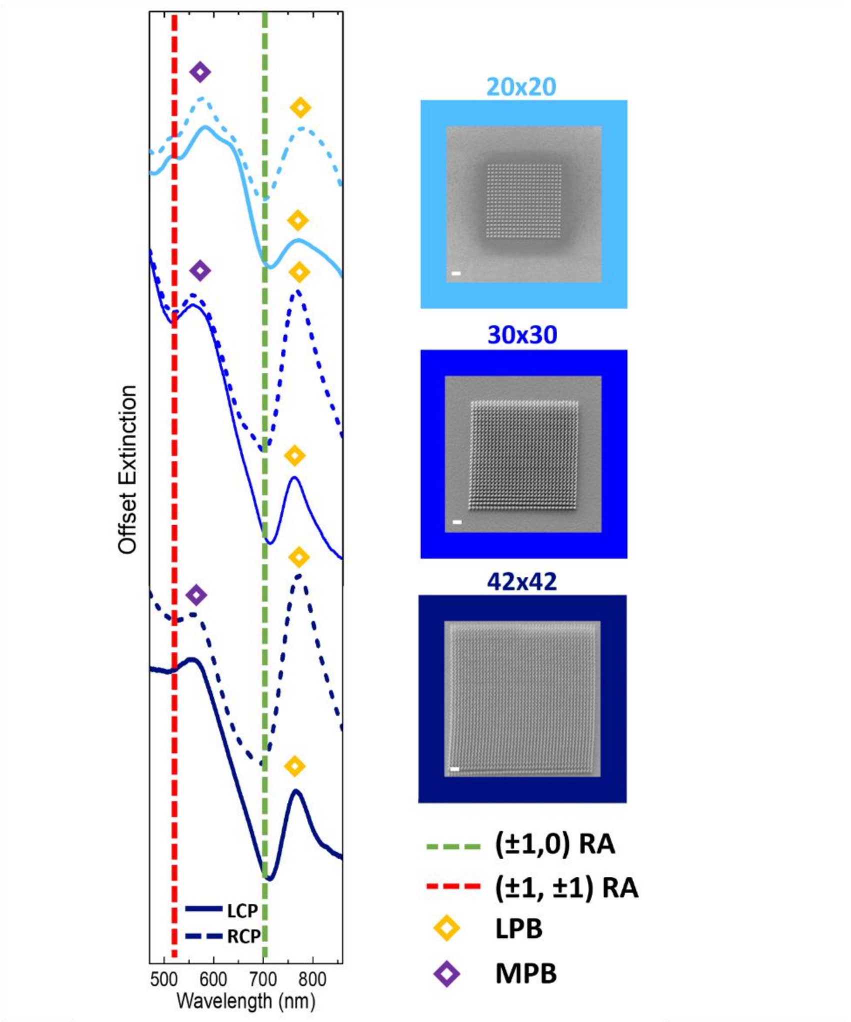


**Figure S1.** Comparison between the left and right-handed circularly polarized extinction spectra derived from transmission measurements of helix array (LP 460 nm) in oil (n=1.518) with different number of elements: 42x42 elements corresponding to a patterned area of 360 $\mu m^2$ (blue), 30x30 elements corresponding to a patterned area of 225 $\mu m^2$ (blue), and 20x20 elements corresponding to a patterned area of 100 $\mu m^2$ (light blue) showed in the left panel SEM image with scale bar 2 μm. The symbols with the same colors indicate similar spectral features (displayed in the legend) observed in both arrays.

## S2. Effect of Lattice Period

Circularly polarized extinction spectra have been extracted from the central pixel of the measured extinction maps of the arrays of 30x30 elements with four different lattice periods (400nm, 430nm, 460nm, 490nm) displayed in the main text (figure 2). In periodic arrays of plasmonic chiral nanostructures, the chiral LSPRs can couple to the diffractive lattice modes of the array, generating the c-SLRs. This results in a peak in the extinction spectra (circle in figure S2a) preceded by a dip related to the linear dispersion of the (±1,0) RAs. The extinction intensity increases with LP, and it is found to be higher for RCP, when incident polarization matches the same handedness of the helices, while is lower when interacting with the opposite light handedness. When increasing LP, a redshift of the spectral features and a progressive spectral narrowing is observed. For LP 460nm and LP 490nm, two additional modes are observed, corresponding to the coupling with (±1,±1)RAs. This confirms that moving towards configurations different from the zero-detuning strong coupling regime, the CD increases [5].

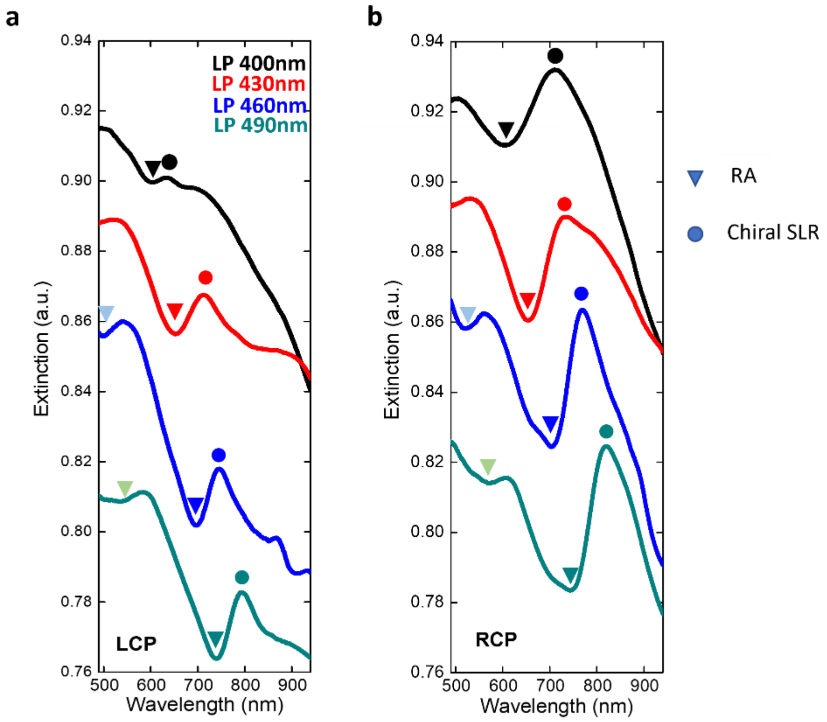


**Figure S2.** a-b. LCP and RCP extinction spectra recorded for arras of 30x30 elements and with different LPs in oil environment. The maxima are attributed to the c-SLRs spectral peaks (indicated by the circles), while the dips indicate the diffractive (±1,0) RAs (represented by the triangle). In the arrays with LP=460nm and LP=490nm, the lightest triangles indicate the (±1,±1) RAs.

## S3. Circularly Polarized Light Extinction Maps

In figure S3 we display the measured LCP, RCP extinction maps and the retrieved CD. The c-SLR onset is evident for both the two incident polarizations, but with different intensity due to a more efficient dipole excitation in RH structure from RCP incident light. This is confirmed in the CD maps where chiral SLR signatures can also be observed. Their angular dispersion in the maps can be clearly distinguished confirming the hybrid nature of the c-SLR. In addition to what discussed in the main text, we highlight that the lower polariton branch (UP) mode superimposes with the (±1,±1)RAs generating a new mode splitting ((±1,±1)RAs  at 510 and MP at 560nm) for LP =490 nm.

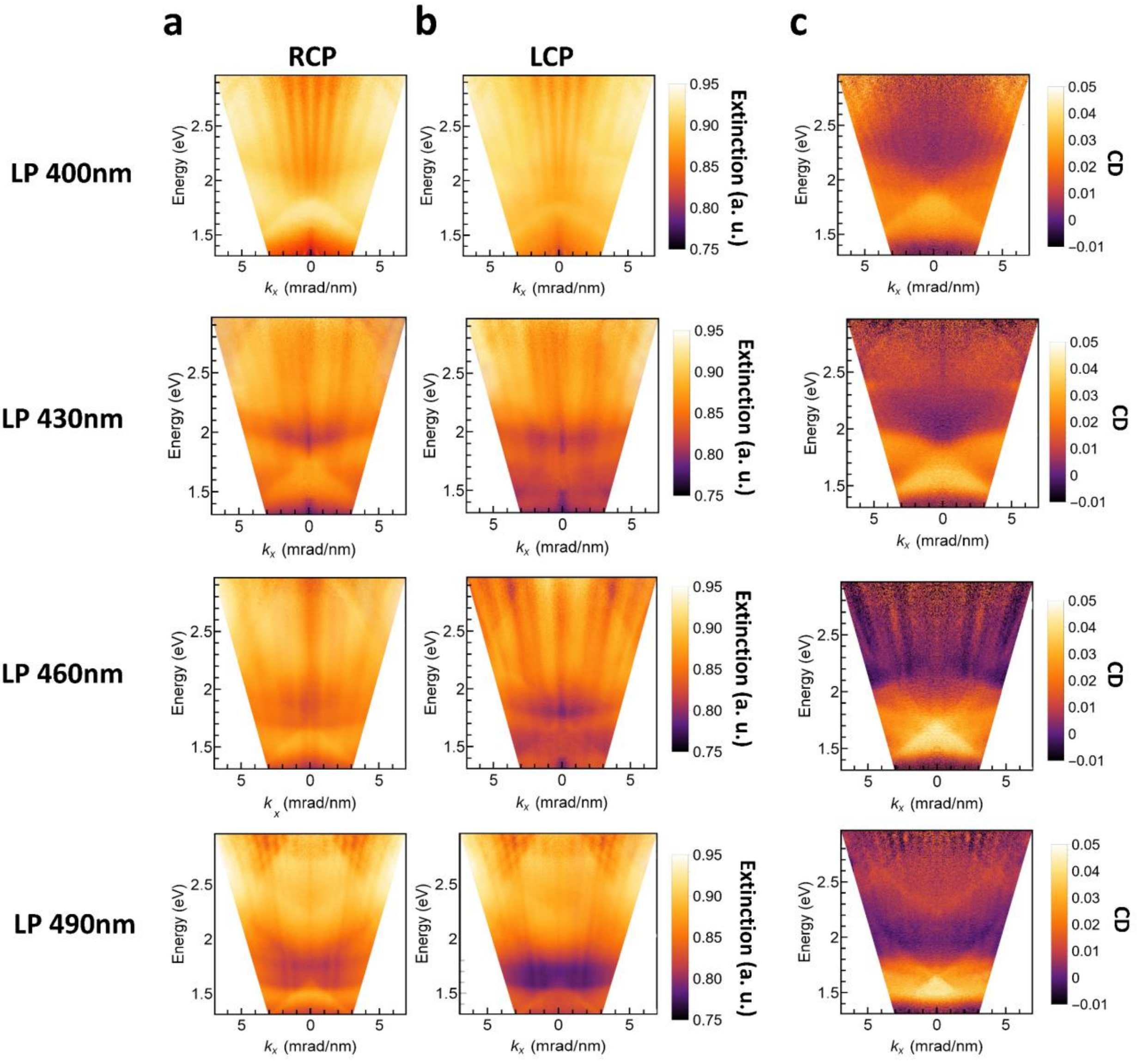


**Figure S3**. a-b. Experimentally measured energy-momentum extinction dispersions for LCP and RCP light for four different lattice periods (400 nm, 430 nm, 460 nm, 490 nm). c. Circular dichroism far-field maps as a function of LP calculated as the difference between RCP and LCP extinctions.

## S4. Numerical simulations of the energy-momentum far field extinction dispersions

The simulated extinction far-field maps of figure S4 are retrieved under interaction with LCP and RCP light in oil environment for different lattice periods: 400nm, 430nm, 460nm, 490nm. They present a good agreement with what observed experimentally and reported in the main text. In particular, we observe a similar trend of the c-SLR with the LP variation with the experimental data. RCP extinction maps display higher intensity, suggesting a better spectral overlap when matching the polarization and the structural handedness, according to experimental data.

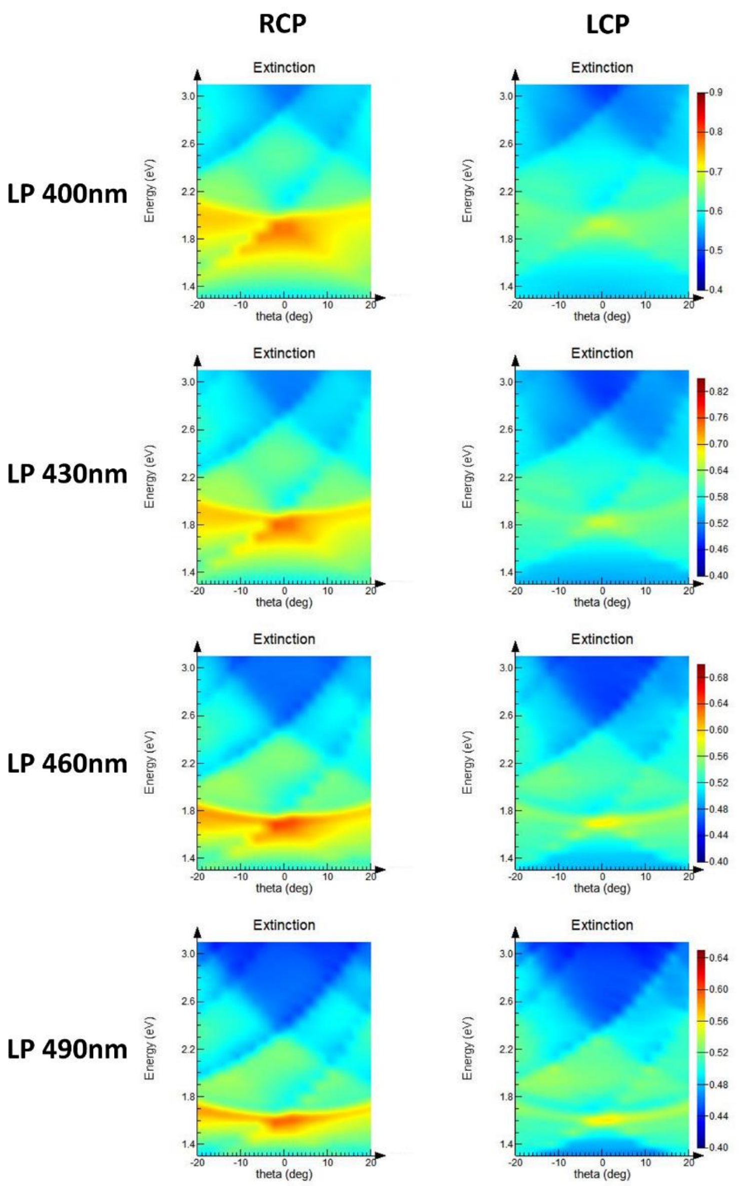


**Figure S4.** RCP and LCP simulated far-field extinction maps evaluated in oil environment for different LPs.

## S5. Calculated Hopfield Coefficients

Figure S5 a,b,c display the Hopfield coefficients for the upper, lower and middle branches, respectively, referred to RCP incident light condition discussed in the main text (figure 3 a). The plots describe how the photonic/plasmonic fraction of the hybrid mode changes with LP.

The Hopfield coefficients (α) can be calculated as in [6]:

$$\alpha = \frac{1}{\sqrt{2}} \frac{\frac{\Delta}{2} + \Omega}{\sqrt{\left(\frac{\Delta}{2}\right)^2 + g^2 + \frac{\Omega\Delta}{2}}}$$

Where Δ is the detuning at a given lattice k-vector, $\Omega = \sqrt{\left(\frac{\Delta}{2}\right)^2 + g^2}$ and g is the coupling coefficient, that is half the separation between the lower and upper branches at zero detuning.

In the lower branch, for low LPs (<340nm) the hybrid mode is mostly plasmon-like. Increasing LP, the plasmon mode fraction decreases and the (±1,0) diffractive order mode weight becomes predominant. At

LP = 340 nm, that is the zero detuning condition, the plasmonic and the (±1,0) diffractive order fractions are the same.

Analogously, in the upper branch(figure S5b) for low LPs (<440nm) the mode is almost pure diffractive orders (DO)-like, while increasing LP, the diffractive order mode fraction decreases and the plasmon fraction increases; for higher LP, the latter value becomes predominant. The middle branch composition (Figure S5c) is more complex since it is weighted over the plasmonic and the different diffractive orders contributions.

Under LCP illumination, the calculated extinction dispersion (figure S5d) shows a plasmon energy extracted at 2.9eV (indicated by a solid line), while the uncoupled dispersions are represented by the black dashed lines. The solid white lines plot the fitting of the simulated peaks through the three coupled-oscillator model given by equation 2. The coupling strength gave values of $g_{[\pm1,0]} \sim 270$meV and $g_{[\pm1,\ \pm1]} \sim 170$meV. For the Hopefield coefficients, in all the calculated branches a behaviour similar to RCP excitation is observed (Figure S5e).

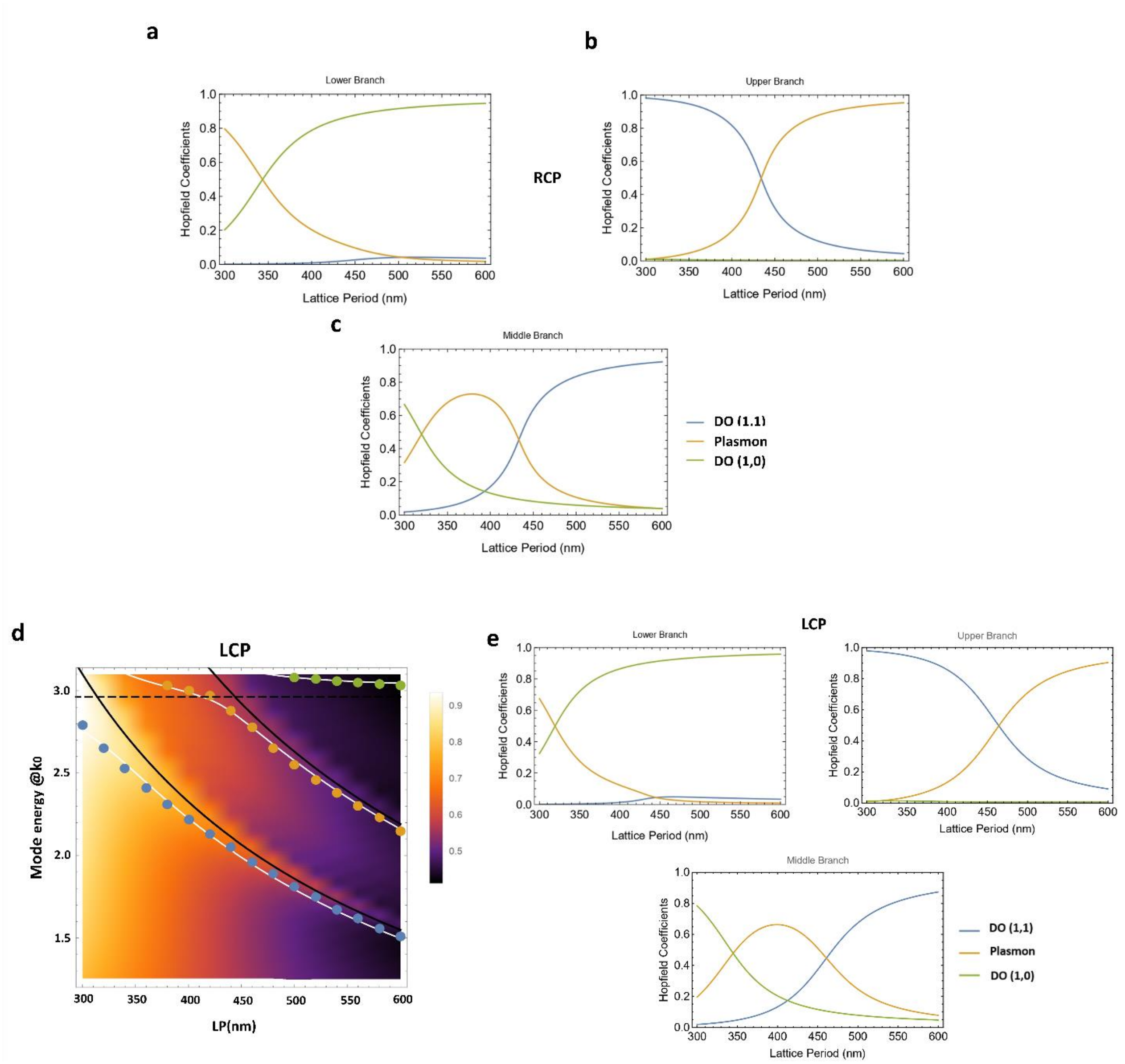


**Figure S5.** Hopefield coefficients calculated from RCP extinction maps for the lower (a), upper (b), and middle (c) branches. d) Extinction dispersion at normal incidence for left-handed circularly polarized light in water environment obtained from numerical simulations. e) Hopfield coefficients for LCP retrieved from the simulated extinction dispersion.

**S6. Q-factor**

The Q-factor of figure S6 was calculated from the experimental spectra acquired in water environment (n=1.3334) as:

$Q = \lambda/\Delta\lambda$,

where λ corresponds to the spectral peak of c-SLRs, and Δλ is the full width at half maximum derived by the Gaussian fit of the SLR spectra. The measured Q-factor shows a clear increase with the lattice period, caused by a reduction of plasmonic content. It should be noted that the material composition of NH fabricated by FIBID is not purely metallic, thus explaining the low absolute values of measured c-SLR Q-factor.

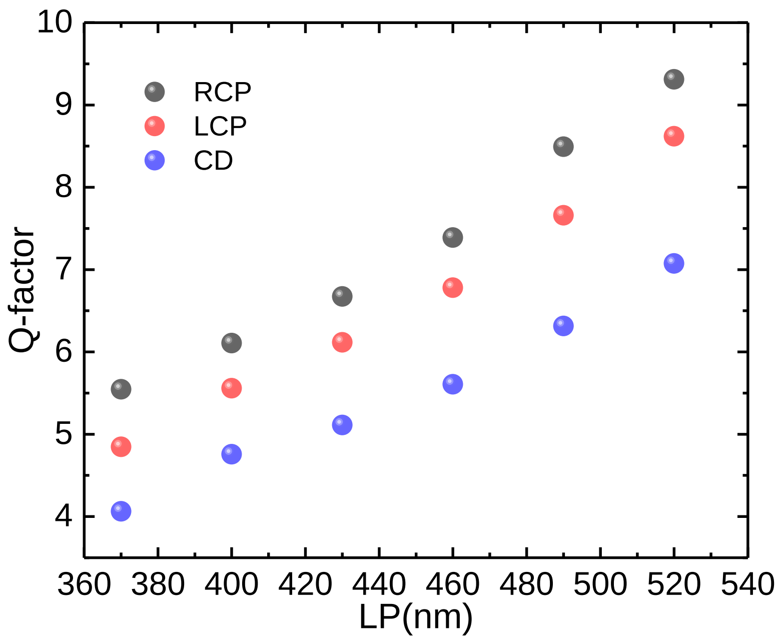


**Figure S6.** Q- factor values calculated for LCP, RCP and CD extinction measured in water environment (n=1.3334) for different LPs.

**S7. CD sensitivity to refractive index variations**

The CD spectra of the samples with different lattice periods were collected in known refractive index (RI) environment. We considered glycerol–water mixtures with varying concentration from 0 to 20% (corresponding to a refractive index range between 1.333 and 1.358)[7]. All the spectra red-shift and, in particular, we focused on the shift of CD maxima, shown in the panels of figure S7, which were used to calculate the sensitivity values in nm/RIU shown in the main text, figure 4a.

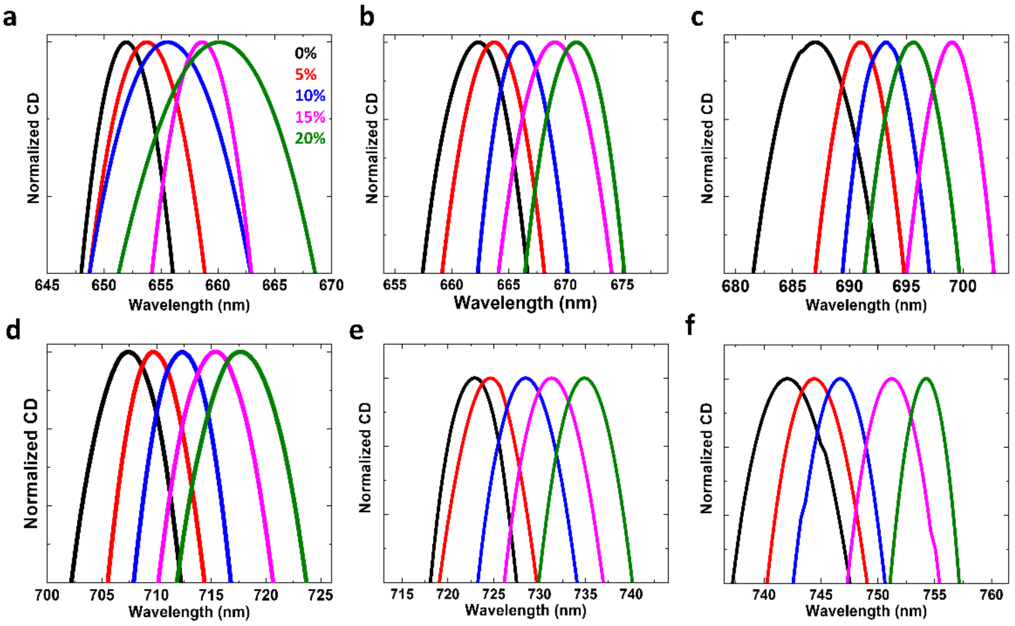


**Figure S7** a-f. CD spectra of the NH arrays as a function of the surrounding refractive index environment (0%, 5%, 10%, 15% 20% of glycerol in water solution), zoomed at the relative resonance peaks measured for different lattice periods: a)LP 370nm, b)LP 400nm, c) LP 430nm, d) LP 460nm, e) c) LP 490nm, f) LP 520nm.

## S8 Calculated Field enhancement

We have performed simulations of the field enhancement (M=E/E0), as a function of the LP, averaged at three different points along the nanohelix z-evolution, in particular at the bottom, center and top side. We found that the M value is maximized for LP=490nm, corresponding to a plasmonic fraction of 0.1.

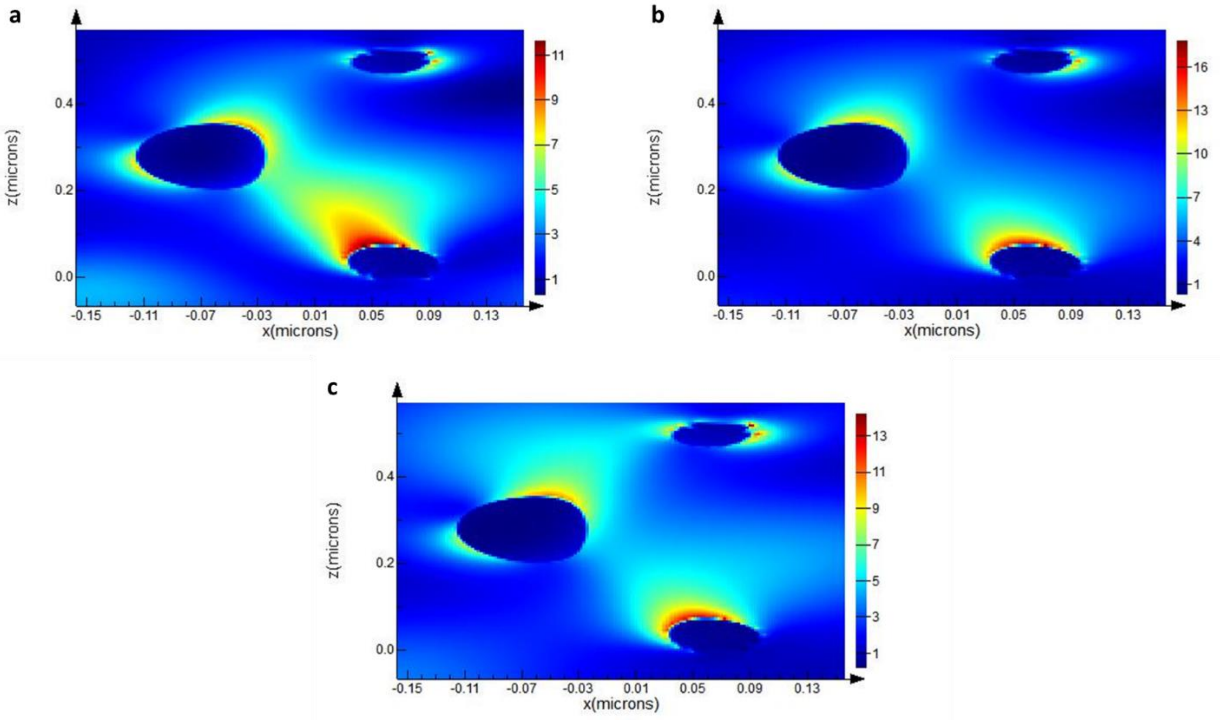


**Figure S8**. Calculated field enhancement as a function of LP= a) 420 nm, b) 490 nm, c) 550 nm.

References


[1] L. Zundel, A. Manjavacas, *J. Phys. Photonics* **2018**, *1*, 15004.

[2] S. R. K. Rodriguez, M. C. Schaafsma, A. Berrier, J. Gómez Rivas, *Phys. B Condens. Matter* **2012**, *407*, 4081.

[3] V. I. Zakomirnyi, S. V Karpov, H. Ågren, I. L. Rasskazov, *J. Opt. Soc. Am. B* **2019**, *36*, E21.

[4] E. S. A. Goerlitzer, R. Mohammadi, S. Nechayev, K. Volk, M. Rey, P. Banzer, M. Karg, N. Vogel, *Adv. Mater.* **2020**, *32*, 2001330.

[5] G. Song, J. Guo, G. Duan, R. Jiao, L. Yu, *Nanotechnology* **2020**, *31*, 345202.

[6] M. I. Vasilevskiy, D. G. Santiago-Pérez, C. Trallero-Giner, N. M. R. Peres, A. Kavokin, *Phys. Rev. B* **2015**, *92*, 245435.

[7] N.-S. Cheng, *Ind. Eng. Chem. Res.* **2008**, *47*, 3285.
